# A long-acting β_2_-adrenergic agonist increases the expression of muscarine cholinergic subtype-3 receptors by activating the β_2_-adrenoceptor cyclic adenosine monophosphate signaling pathway in airway smooth muscle cells

**DOI:** 10.3892/mmr.2015.3307

**Published:** 2015-02-05

**Authors:** YUAN-HUA LIU, SONG-ZE WU, GANG WANG, NI-WEN HUANG, CHUN-TAO LIU

**Affiliations:** 1 Department of Respiratory and Critical Care Medicine, West China Hospital, Sichuan University, Chengdu, Sichuan 610041, P.R. China; 2 Department of Respiratory and Critical Care Medicine, The First Affiliated Hospital, Zhengzhou University, Zhengzhou, Henan 450052, P.R. China; 3 Pneumology Group, Department of Integrated Traditional Chinese and Western Medicine, West China Hospital, Sichuan University, Chengdu, Sichuan 610041, P.R. China

**Keywords:** airway smooth muscle cells, β_2_-adrenoceptor agonists, bronchoprotection, formoterol, muscarine cholinergic subtype 3 receptor, phospholipase C-β_1_

## Abstract

The persistent administration of β_2_-adrenergic (β_2_AR) agonists has been demonstrated to increase the risk of severe asthma, partly due to the induction of tolerance to bronchoprotection via undefined mechanisms. The present study investigated the potential effect of the long-acting β_2_-adrenergic agonist, formoterol, on the expression of muscarinic M3 receptor (M_3_R) in rat airway smooth muscle cells (ASMCs). Primary rat ASMCs were isolated and characterized following immunostaining with anti-α-smooth muscle actin antibodies. The protein expression levels of M_3_R and phospholipase C-β_1_ (PLCβ_1_) were characterized by western blot analysis and the production of inositol 1,4,5-trisphosphate (IP_3_) was determined using an enzyme-linked immunosorbent assay. Formoterol increased the protein expression of M_3_R in rat ASMCs in a time- and dose-dependent manner, which was significantly inhibited by the β_2_AR antagonist, ICI118,551 and the cyclic adenosine monophosphate (cAMP) inhibitor, SQ22,536. The increased protein expression of M_3_R was positively correlated with increased production of PLCβ_1_ and IP_3_. Furthermore, treatment with the glucocorticoid, budesonide, and the PLC inhibitor, U73,122, significantly suppressed the formoterol-induced upregulated protein expression levels of M_3_R and PLCβ_1_ and production of IP_3_. The present study demonstrated that formoterol mediated the upregulation of M_3_R in the rat ASMCs by activating the β_2_AR-cAMP signaling pathway, resulting in increased expression levels of PLCβ_1_ and IP_3_, which are key to inducing bronchoprotection tolerance. Administration of glucocorticoids or a PLC antagonist prevented formoterol-induced bronchoprotection tolerance by suppressing the protein expression of M_3_R.

## Introduction

Asthma is a chronic airway inflammatory disease with increasing prevalence worldwide (1,2). The airways of patients with asthma are hyper-responsive to exercise, allergens and contractile agents, including histamine and acetylcholine (ACh) (3,4). Due to bronchodilatation and bronchoprotection, which prevent bronchoconstriction, long-acting β_2_-adrenergic agonists (LABAs), including salmeterol and formoterol, have been widely combined with inhaled corticosteroids to treat patients with asthma that respond poorly to corticosteroid-only based therapies (5). However, LABAs alone increase the risk of asthma-associated mortality (6), possibly due to increased bronchial hyper-responsiveness (7), severe exacerbation of asthmatic symptoms (8) or tolerance to bronchodilation and bronchoprotection (9–11).

Previous studies have demonstrated that adenylyl cyclase stimulation results in the subsequent activation of cyclic adenosine monophosphate (cAMP)/protein kinase A (PKA) associated with LABA-induced rapid bronchodilatation (12,13). By contrast, contractile agonists, including ACh, were revealed to initiate bronchoconstriction as a result of G-protein-coupled muscarinic M3 receptors (M_3_R) binding to airway smooth muscle cells, resulting in the subsequent activation of phospholipase C (PLC) and the production of inositol 1,4,5-trisphosphate (IP_3_) (12,14). Chilvers *et al* reported that pretreatment with salmeterol significantly inhibits histamine-stimulated accumulation of IP_3_ (15) and McGraw *et al* demonstrated that transgenic mice overexpressing airway smooth muscle β_2_-adrenoceptor (β_2_AR) agonists significantly increase the expression of PLC-β_1_ compared with that of wild-type mice (14), suggesting that the sustained activation of β_2_AR induces the PLCβ_1_-IP_3_ signaling pathway via mechanisms that remain to be elucidated.

The present study investigated the effects of formoterol on the expression of M_3_R and the downstream signaling events leading to bronchoprotection tolerance in rat airway smooth muscle cells (ASMCs).

## Materials and methods

### Reagents

Formoterol, (SQ22,536), a cAMP antagonist, ICI118,551, a β_2_AR antagonist, H89, PKA antagonist, budesonide, a glucocorticoid and U73,122, an PLC inhibitor, were purchased from Tocris Bioscience (Bristol, UK), and forskolin, a cAMP stimulator, and ACh were purchased from Sigma-Aldrich (St. Louis, MO, USA). Dulbecco’s modified Eagle’s medium (DMEM), fetal bovine serum (FBS) and 0.25% trypsin, containing ethylenediamine tetraacetic acid, were purchased from Gibco Life Technologies (Carlsbad, CA, USA). Rabbit polyclonal anti-α-smooth muscle actin antibody (cat. no. ab5694; 1:100 for immunocytochemistry and 1:2,000 for western blot analysis) and anti-muscarinic ACh receptor M_3_ antibody (cat. no. ab41169; 1:100 for immunocytochemistry and 1:500 for western blot analysis) were purchased from Abcam (Cambridge, UK). A mouse polyclonal anti-rat anti-PLCβ_1_ antibody (cat. no. 610924; 1:1,000) was purchased from Becton Dickinson (Dublin, Ireland). Mouse anti-β-actin and fluorescein isothiocyanate-conjugated anti-rabbit immunogobluin (Ig)G (cat. no. ZF-0311; 1:100) antibodies were purchased from Zhongshan Golden Bridge Biological Technology Co. (Beijing, China). Horseradish peroxidase-conjugated goat anti-rabbit IgG (1:20,000) and goat anti-mouse IgG (1:20,000) secondary antibodies were obtained from Pierce (Rockford, IL, USA). The IP_3_ enzyme-linked immunosorbent assay (ELISA) kit was purchased from Cusabio Biotech Co., Ltd. (Wuhan, China).

### Primary rat ASMC cultures

Male Wistar rats (8 weeks old; 150±50g) were provided by the Animal Center of West China Hospital, (Sichuan University, Chengdu, China). The rats were housed under specific-pathogen-free conditions at 25°C and maintained on a 12-h light/dark cycle, with access to food and sterile water *ad libitum*. A total of 52 rats were injected (i.p.) with 10% chloral hydrate to anesthetize them, and then they were sacrificed by cervical vertebra dislocation. Primary rat ASMC cultures were prepared, as previously described (16). Briefly, the trachea of each rat was excised, minced and the cells were allowed to adhere to the culture flasks for 3 h. Fresh culture medium (DMEM+FBS) was subsequently added and the cells were grown to confluency (density, 80 cells at x200 high-power lens) in an incubator at 37°C with 5% CO_2_. The cultured cells were passaged following trypsinization (0.05%). ASMCs passaged three times were immunostained with anti-α-smooth muscle actin antibodies. Cells between passages four and six, which were >80% confluent, were used for subsequent experiments. The present study was approved by the Biomedical Research Ethics Committee at West China Hospital (Sichuan University, Chengdu, China).

### Experimental procedures

ASMCs (density, 80 cells at ×200 high-power lens) were incubated in the presence of various concentrations of formoterol (10^−4^, 10^−5^, 10^−6^ or 10^−7^ mmol/l) for 1, 3, 6, 12, 24 and 48 h at 37°C with 5% CO_2_. The addition of the respective antagonists were performed for 2 h at the following concentrations: 10^−5^ mmol/l ICI118,551, 10^−4^ mmol/l SQ22,536 or 10^−5^ mmol/l H89 for 24 h prior to treatment with 10^−5^ mmol/l formoterol. For cAMP stimulation, the cells were incubated with 10^−5^ mmol/l forskolin for 24 h at 37°C with 5% CO_2_. When multiple compounds were used, the cells were treated with 10^−5^ mmol/l formoterol in the presence of 10^−4^ mmol/l budesonide or 10^−5^ mmol/l U73,122 for 24 h at 37°C with 5% CO_2_. The cells in the control group were cultured in DMEM+FBS only, at 37°C with 5% CO_2_. To observe the effect of formoterol on bronchoconstriction prevention (bronchoprotection), the cells were first stimulated with the contractile agonist ACh (10^−4^ mmol/l) for 15 min, followed by the above mentioned treatments and analyzed by western blot analysis to determine the protein expression levels of M_3_R and PLCβ_1_, in addition to determining the expression of IP_3_ by ELISA.

### Immunocytochemistry

The cells (density, 80 cells at ×200 high-power lens) were fixed with 4% paraformaldehyde, blocked with goat serum (10%; Merck Millipore, Boston, MA, USA) and probed with primary antibodies specific to α-smooth muscle actin (1:100) or M_3_R (1:100) overnight at 4°C, followed by incubation with secondary antibody (1:100) at 37°C for 1 h. The nuclei were stained with 4′,6-diamidino-2-phenylindole (Invitrogen, Carlsbad, CA, USA) for 5 min at room temperature. Images were captured using a confocal laser-scanning microscope (IX71-F22FL/PH, Olympus, Tokyo, Japan).

### Western blot analysis

The protein expression levels of M_3_R and PLCβ_1_ were measured by western blot analysis. The total cellular protein was extracted using radioimmunoprecipitation assay lysis buffer (1% Triton-X, 0.5% sodium deoxychlate, 0.1% SDS; Sangon Biotech, Shanghai, China), quantified using a bicinchoninic acid assay (Boster, Wuhan, China) and a Model 680 spectrophotometer (Bio-Rad Laboratories, Inc., Hercules, CA, USA) and the total protein concentration was adjusted to 0.8 *μ*g/*μ*l. Equal quantities of protein were subjected to 5% sodium dodecyl sulphate polyacrylamide gel electrophoresis (12.6% separation gel for M_3_R, β_2_AR and β-actin; 10% separation gel for PLCβ1; Sigma-Aldrich) and subsequently transferred onto polyvinylidene fluoride membranes (Merck Millipore). The membranes were blocked for 1 h with Tris-buffered saline containing 0.05% Tween-20 (TBST; Boster) and 5% goat serum (Boster), for M_3_R blots or with 5% (w/v) non-fat milk for the PLCβ_1_ and β-actin blots. The membranes were subsequently incubated with primary antibodies against anti-M_3_R (1:500), anti-PLCβ_1_ (1:1,000) or anti-β-actin (1:2,000) at 4°C overnight. Following incubation, the membranes were washed three times with TBST for 10 min and incubated with anti-rabbit (1:20,000) or anti-mouse (1:20,000) secondary antibodies for 1 h at room temperature. The membranes were subsequently washed and the blots were visualized using a Bio-Rad Gel Doc™ XR+ Imaging system and the band densities were quantified using Quantity One software (Bio-Rad Laboratories, Inc.).

### ELISA

The levels of IP_3_ were determined using an IP3 ELISA kit (Cusabio Biotech Co., Ltd, Wuhan, China), according to the manufacturer’s instructions. The ASMC culture medium was removed and the cells were incubated with 0.1 mmol/l HClO_4_ for 20 min. The cells were centrifuged at 170 × g for 15 min at room temperature and the supernatant was collected for analysis. An anti-IP_3_ detection antibody was added and incubated at 37°C for 60 min, followed by the addition of substrate solution for 15 min at 37°C. The reaction was terminated following the addition of stop solution and the plates were read at an absorbance of 450 nm using a Model 680 spectrophotometer (Bio-Rad Laboratories, Inc.). The effect of formoterol on the expression of IP_3_ was determined using the following formula: Inhibition of ACh-induced IP_3_ accumulation (%) = (IP_3_ levels in the control group - IP_3_ levels in the treatment group) / IP_3_ levels in the control group × 100%.

### Statistical analysis

Data are expressed as the mean ± standard deviation and the differences between groups were analyzed using analysis of variance or non-paired Student’s t-test if the continuous variables were not normally distributed. The associations between M_3_R and IP_3_ or PLCβ_1_ were determined using a linear regression model. All statistical analyses were performed using SPSS 17.0 (SPSS, Inc., Chicago, IL, USA). P<0.05 was considered to indicate a statistically significant difference.

## Results

### Characterization of rat ASMCs

**Figure 1:**
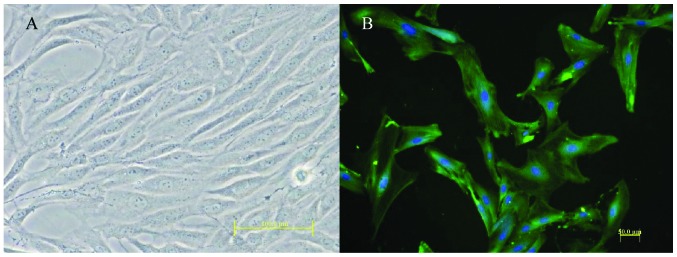
Primary cultures of rat AMSCs. (A) Confluent ASMCs visualized under phase-contrast microscopy (magnification, ×200). (B) ASMCs assessed by immunocytochemistry following incubation with an anti-α-smooth muscle actin antibody. Nuclei were stained using 4′,6-diamidino-2-phenylindole (magnification, ×200). ASMCs, airway smooth muscle cells.

The confluent rat ASMCs were relatively homogeneous, with a hill-and-valley pattern (Fig. 1A). Anti-α-smooth muscle actin (a SMC-specific marker) was diffusely distributed within the cytoplasm and the purification of ASMCs between passages four and six was confirmed to be >95% (Fig. 1B).

### Formoterol upregulates the protein expression of M_3_R in ACh-stimulated rat ASMCs in a time- and dose-dependent manner

**Figure 2:**
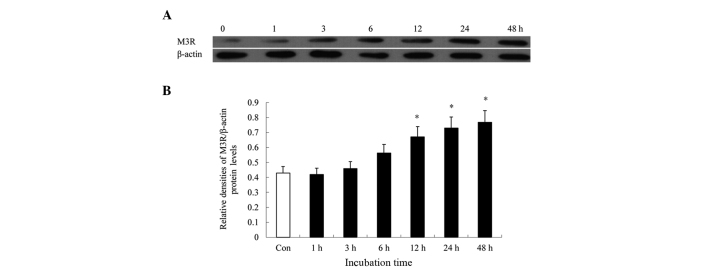
Formoterol upregulates the expression of M_3_R. (A) Protein expression in airway smooth muscle cells treated with or without 10^−5^ mmol/l formoterol at the indicated time points were examined by western blot analysis. (B) Protein expression of M_3_R was determined by densitometry and was normalized to the β-actin control. The data are expressed as the mean ± standard deviation from three independent experiments (^*^P<0.05, compared with the untreated 0 h group). M_3_R, muscarinic M_3_ receptor; Con, control.

**Figure 4:**
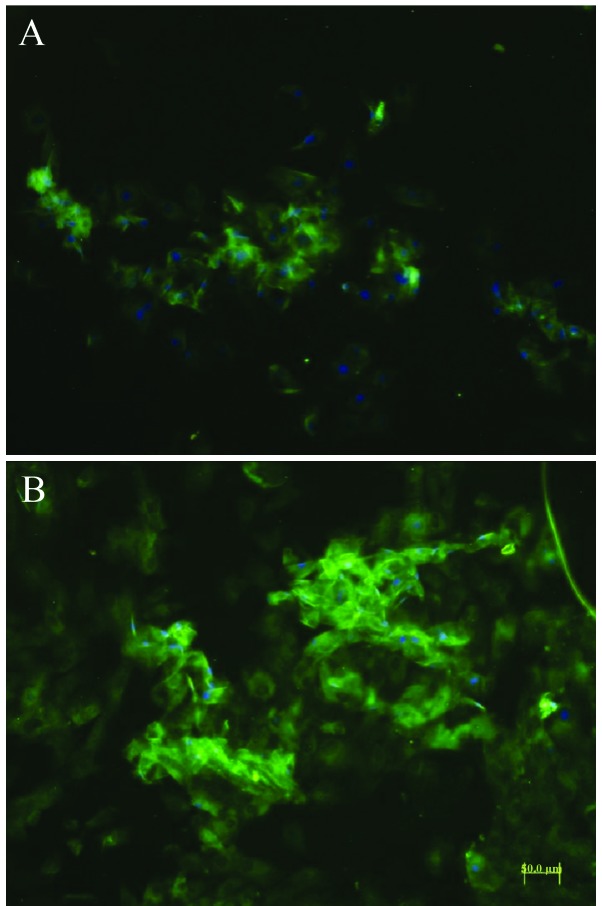
Distribution of M_3_Rs in rat airway smooth muscle cells. The cells were treated with 10^−5^ mmol/l formoterol for (A) 1 h or (B) 24 h. The expression of M_3_R was evaluated by immunostaining using an anti-M_3_R antibody following stimulation with acetylcholine for an additional 15 min. Nuclei were stained with 4′,6-diamidino-2-phenylindole (magnification, ×100). M_3_R, muscarinic M_3_ receptor.

Treatment with formoterol increased the expression of M_3_R in a time- and dose-dependent manner in the rat ASMCs, with a maximal induction observed at 24 h in the presence of 10^−5^ and 10^−4^ mmol/l formoterol (Figs. 1 and 2). The clinical concentration of plasma formoterol is significantly lower than 10^−4^ mmol/l (17), therefore, 10^−5^ mmol/l formoterol was selected for the subsequent experiments. The immunocytochemical analysis demonstrated that the expression of M_3_R was significantly increased and predominantly located in the cellular membrane (Fig. 4). These results suggested that formoterol upregulated the protein expression of M_3_R in rat ASMCs.

### Formoterol regulates the expression of M_3_R through the β_2_AR-cAMP signaling pathway

**Figure 5:**
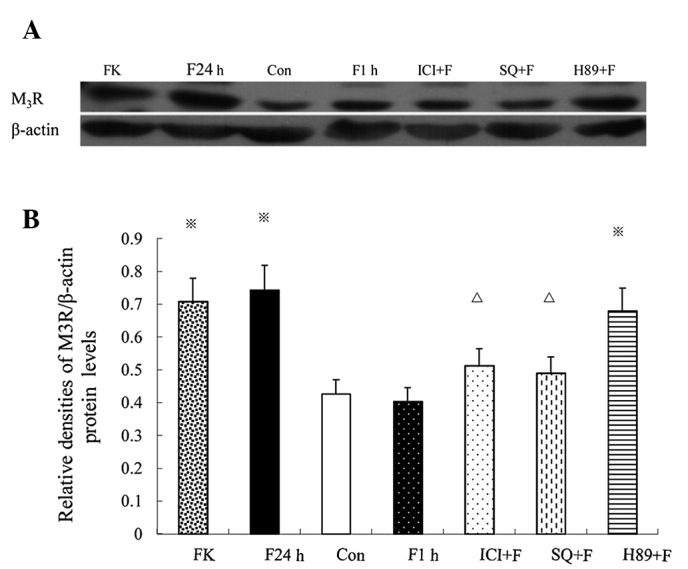
Formoterol regulates the expression of M3R by mediating signaling via the β2AR-cAMP signaling pathway. (A) Rat airway smooth muscle cells were randomly divided into seven groups. The cells were treated with formoterol (10^−5^ mmol/l) for 1 h or 24 h. The cells stimulated with cAMP were treated with 10^−5^ mmol/l forskolin for 24 h. Inhibition of the β2AR-cAMP-protein kinase A was performed by pretreating the cells with 10^−5^ mmol/l ICI118,551, 10^−4^ mmol/l SQ22,536 or 10^−5^ mmol/l H89 for 24 h. These treatment groups and the control group were subsequently treated with 10^−5^ mmol/l formoterol for 2 h. The protein expression of M3R in the rat airway smooth muscle cells was determined by western blot analysis following acetylcholine stimulation for 15 min. (B) Expression of M3R was normalized to the β-actin control. The data are expressed as the mean ± standard deviation from three independent experiments (^*^P<0.01, compared with the 1 h incubation group; ^Δ^ P<0.05, compared with the 24 h formoterol treatment group). F1h, 1 h formoterol treatment; F24h, 24 h formoterol treatment; FK, forskolin; ICI+F, formoterol+ICI118,551; SQ+F, formoterol+SQ22,536; H89+F, formoterol+H89; Con, control; M_3_R, muscarinic M_3_ receptor.

Pre-treatment with the ICI118,551 β_2_AR antagonist or the SQ22,536 cAMP inhibitor significantly antagonized the formoterol-induced expression of M_3_R (P<0.01; Fig. 5). However, the H89 PKA inhibitor had no effect on the formoterol-regulated expression of M_3_R (P>0.05). As expected, the forskolin cAMP stimulator caused similar effects as formoterol with respect to the protein expression of M_3_R. The present study demonstrated that 24 h incubation with forskolin significantly increased the protein expression of M_3_R (P<0.01), compared with the control and compared with levels 24 h after treatment with formoterol. These results suggested that formoterol induced the expression of M_3_R through the β_2_AR-cAMP signaling pathway.

### Formoterol-induced upregulation of M_3_R is associated with increased expression levels of PLCβ_1_ and IP_3_

**Figure 6:**
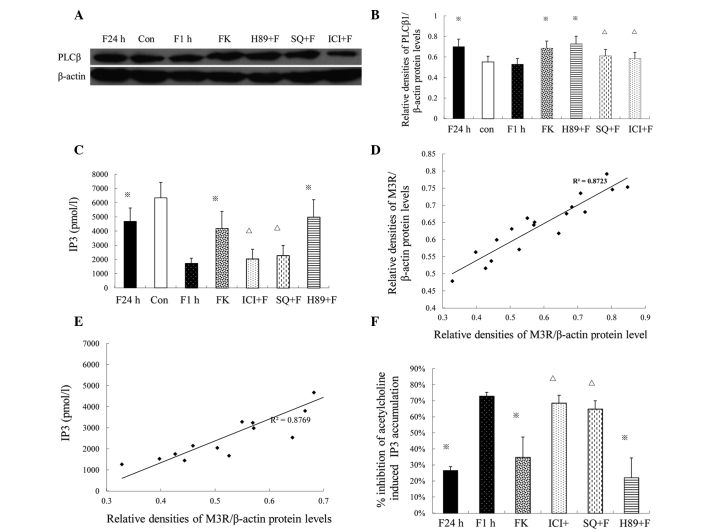
Formoterol-induced upregulation of the expression of M_3_R is associated with increased expression levels of PLCβ1 and IP3. (A) Rat ASMCs were randomly divided into seven groups and treated with formoterol with or without inhibitors and for different durations. Untreated cells were used as a control. The protein expression of rat ASMCs PLCβ1 was determined by western blot analysis. (B) Protein expression of PLCβ1 was normalized to the β-actin control. The data are expressed as the mean ± standard deviation from three independent experiments. (C) Expression of IP3 was determined by ELISA. (D and E) Correlations between the expression levels of PLCβ1 or IP3 and the expression of M_3_R were determined using linear regression. (F) Inhibitory rate of acetylcholine-induced IP3 accumulation (^*^P<0.01, compared with the 1 h incubation group; ^Δ^ P<0.05, compared with the 24 h formoterol only treatment group). F1h, 1 h formoterol treatment; F24h, 24 h formoterol treatment; FK, forskolin; ICI+F, formoterol+ICI118,551; SQ+F, formoterol+SQ22,536; H89+F, formoterol+H89; Con, control; IP3, inositol 1,4,5-trisphosphate; M3R, muscarinic M3 receptor, PLCβ, phospholipase C-β.

The present study demonstrated that formoterol increased the expression of PLCβ_1_ in ACh-stimulated rat ASMCs (Fig. 6A and B). Inhibition of the β_2_AR-cAMP signaling pathway using the ICI118,551 or SQ22,536 antagonists inhibited the formoterol-induced upregulation of PLCβ_1_ (P<0.05). By contrast, no significant difference was observed in the expression of PLCβ_1_ following exposure to the H89 PKA inhibitor in the presence of formoterol (P>0.05). Forskolin had a similar effect on the formoterol-induced expression of PLCβ_1_. In addition, treatment with formoterol for 1 h suppressed the ACh-induced production of IP_3_ by ~72.89±2.29%, compared with the 26.58±2.37% inhibition observed following formoterol exposure for 24 h (Fig. 6E). Similarly, ICI118,551 and SQ22,536 also reduced the expression of IP_3_. Positive correlations were observed between M_3_R and PLCβ_1_ (R^2^= 0.872; P<0.01) and between M_3_R and IP_3_ (R^2^=0.877, P<0.01), as shown in Fig. 6D and E.

### Effects of a glucocorticoid and a PLC inhibitor on the formoterol-induced upregulation of M_3_R

**Figure 7:**
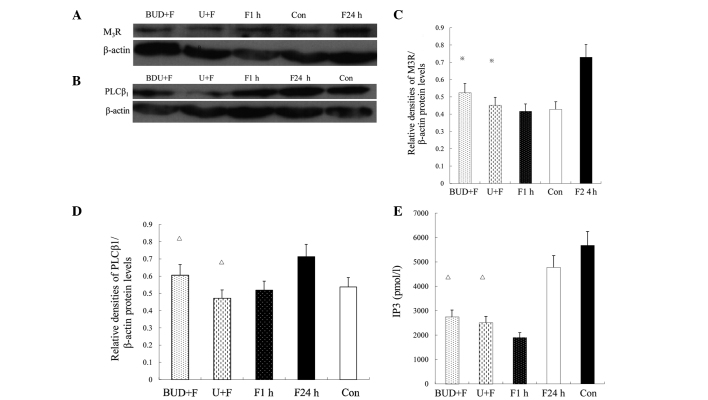
Effects of glucocorticoid and PLC inhibitors on formoterol-induced upregulation of the expression of M3R. Rat ASMCs were randomly divided into five groups and treated as follows: 10^−5^ mmol/l formoterol for 1 or 24 h; 10^−5^ mmol/l formoterol for 24 h in the presence of 10^−4^ mmol/l BUD (glucocorticoid, BUD+F) or 10^−5^ mmol/l U73,122 (PLC inhibitor) or untreated. The expression levels of M3R, PLCβ1 and IP3 in the different treatment groups and the control were determined following a 15 min acetylcholine (10^−4^ mmol/l) stimulation. The protein levels of (A and B) M3R and (C and D) PLCβ1 in rat ASMCs were determined by western blot analysis and were determined by densitometry. The band densities were normalized to the β-actin control. (E) Expression of IP3 was determined by ELISA. The data are expressed as the mean ± standard deviation from three independent experiments. ^Δ^ P<0.05, compared with the 24 h formoterol only group. F1h, 1 h formoterol treatment; F24h, 24 h formoterol treatment; Con, control; IP3, inositol 1,4,5-trisphosphate; M3R, muscarinic M3 receptor, PLC, phospholipase C; BUD, budesonide; U, U73,122.

The combined treatment of budesonide and formoterol significantly reduced the expression levels of M_3_R, PLCβ_1_ and IP_3_ compared with the expression levels observed following treatment with formoterol alone (P<0.05; Fig. 7). In addition, the U73,122 PLC inhibitor significantly decreased the formoterol-induced upregulation of the protein expression levels of M_3_R and PLCβ_1_ and the production of IP_3_ compared with formoterol treatment alone (P<0.05).

## Discussion

Emerging evidence has demonstrated that prolonged administration of LABAs increases the risk of asthma-associated mortality (6) or can seriously exacerbate asthmatic symptoms (8), possibly due to increased bronchial hyper-responsiveness (7) and bronchodilator and broncho-protection tolerance (9–11) The β_2_AR, fenoterol, induces the upregulation of G-protein-coupled neurokinin receptors and H1 histamine receptors in ASMCs (18,19) This suggests that β_2_AR may lead to increased bronchial responsiveness and bronchodilator tolerance by upregulating the expression of G-protein-coupled receptors. However, bronchoprotection gradually decreases in the presence of sustained administration of LABAs via mechanisms, which remain to be elucidated.

M_3_R is a G-protein-coupled receptor predominantly distributed on the membrane surface of ASMCs. In the present study the effects of formoterol, a widely used LABA, on the expression of M_3_R was investigated in rat ASMCs. Formoterol upregulated the expression of M_3_R for at least 48 h, however, the long-term effects of formoterol were not evaluated due to the rapid proliferation of ASMCs *in vitro*. It has been suggested that stimulation of β_2_AR activates intracellular adenyl cyclase, which catalyzes the conversion of ATP to cAMP, which in turn increases the activity of PKA associated with altered intracellular Ca^2+^ homeostasis and results in bronchodilation (12). Treatment with the ICI118,551 β_2_AR antagonist, SQ22,536 cAMP antagonist or H89 PKA antagonist demonstrated that the β_2_AR-cAMP signaling pathway contributed to the formoterol-mediated upregulation of M_3_R via a PKA-independent mechanism. Consistent with these results, it was previously demonstrated that prolonged exposure to the cAMP-responding element-binding (CREB) protein and c-Ets1 (LABA) contribute to mucous cell hyper-secretion associated with common respiratory disorders (20), suggesting a role for the β_2_AR-cAMP-CREBs signaling pathway in this process. In addition, β_2_AR agonists increased the cAMP-mediated activation of cGMP-dependent protein kinases leading to smooth muscle relaxation (12,21). cAMP can bind to exchange proteins, which are directly activated by cAMP (Epac) independent of PKA, resulting in the induction of Rap-1-dependent responses in the airway smooth muscles, epithelium and pro-inflammatory immune cells (12,22). However, a previous study revealed that β_2_AR agonists selectively inhibit ASMC migration by interfering with the β_2_AR/PKA signaling pathway and that prolonged treatment with albuterol eliminated the inhibitory effect of β-agonists on ASMC migration (13). This suggested that multiple signaling pathways, including PKA, may be involved in β_2_AR agonist functions. Whether the overexpression of M_3_R from prolonged treatment with formoterol is mediated by a cAMP-responding element-binding protein, through the β_2_AR-cAMP signaling pathway, requires further investigation. In addition, further experiments are required to determine whether downstream signaling proteins, in addition to cAMP, are important in the formoterol-induced overexpression of M_3_R.

McGraw *et al* demonstrated that the expression of PLCβ_1_ is significantly increased in transgenic mice overexpressing airway smooth muscle β_2_AR (14) and Sayers *et al* reported that a β_2_AR agonist upregulated the protein expression of PLCβ_1_ in human ASMCs (23). The present study supported these observations and demonstrated that formoterol exposure increased the protein expression of PLCβ_1_ and production of IP_3_ in the rat ASMCs. In addition, changes to the expression levels of PLCβ_1_ and IP_3_ were positively correlated with the expression of M_3_R. Contractile agonists bind to G-protein-coupled M_3_R and trigger the activation of PLC, resulting in the production of IP_3_, leading to Ca^2+^ release and subsequent airway smooth muscle contraction (12,14). A previous study revealed that salbutamol and salmeterol (short- and LABR) inhibit the histamine-stimulated accumulation of IP_3_ in airway smooth muscle cells (15). The data from the present study demonstrated that 24 h pre-treatment with formoterol significantly reduced the ACh-stimulated production of IP_3_. This inhibitory effect on the accumulation of IP_3_, however, was reduced following pre-treatment with formoterol for 24 h (26.58±2.37%) compared with 1 h (72.89±2.29%). These results demonstrated that short-term pre-exposure of ASMCs to formoterol antagonized the accumulation of IP_3_ induced by ACh and that this effect was attenuated significantly if the pre-exposure duration was extended, suggesting that this may be a mechanism contributing to bronchoprotection tolerance.

The present study also demonstrated that inhibiting the β_2_AR-cAMP signaling pathway significantly downregulated the formoterol-induced expression of M_3_R and inhibited the production of IP_3_. The expression of M_3_R was negatively correlated to the rate at which production of IP_3_ was inhibited, suggesting that M_3_R may be important in bronchoprotection tolerance and that cholinergic antagonists may be used in the potential treatment of patients that respond poorly to LABAs. Furthermore, inhibiting PLCβ_1_ significantly reduced the expression of M_3_R and increased the inhibitory effect of formoterol on the production of IP_3_. These results suggested that the inhibition of PLCβ_1_ may provide a novel strategy for preventing bronchoprotection tolerance. However, other mechanisms, including the functional desensitization of β_2_AR in mast cells, may also have contributed to bronchoprotection tolerance (24,25). In addition, β_2_AR agonists may result in membrane hyperpolarization by activating K^+^ channels (26).

Combined treatment with LABAs and inhaled corticosteroids is a common for patients with poorly controlled asthma, which is associated with improved pulmonary function and asthma control (27,28). The data presented in the present study revealed that glucocorticoids suppressed the formoterol-induced upregulation of M_3_R, reduced the expression of PLCβ_1_ and partially facilitated the formoterol-mediated inhibition of IP_3_ production. These observations suggested that the inhibition of the expression of M_3_R may be important in combination therapies designed to prevent bronchoprotection tolerance. However, these studies were performed *in vitro*, therefore the results require confirmation in experimental animal asthma models and in patients.

In conclusion, the present study demonstrated that formoterol upregulated the protein expression of M_3_R in rat ASMCs following activation of the β_2_AR-cAMP signaling pathway, resulting in an increased expression of PLCβ_1_ and IP_3_, which are critical for mediating bronchoprotection tolerance. Administration of a glucocorticoid or PLC antagonist prevented formoterol-induced bronchoprotection tolerance by suppressing the protein expression of M_3_R.

## Figures and Tables

**Figure 3:**
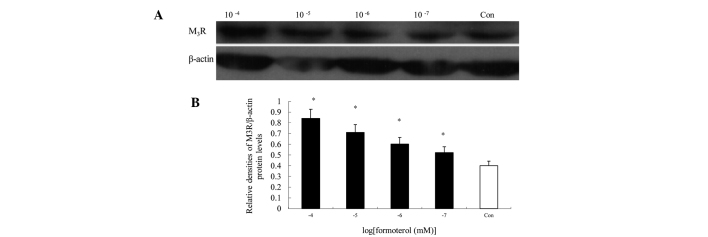
Formoterol upregulates the expression of M_3_R in a dose-dependent manner. (A) Protein extracts were obtained from airway smooth muscle cells treated with increasing concentrations of formoterol for 24 h and the expression of M_3_R was analyzed by western blot analysis. (B) Protein expression of M_3_R was determined by densitometry and was normalized to the β-actin control. The data are expressed as the mean ± standard deviation from three independent experiments (^*^P<0.05, compared with the control). M_3_R, muscarinic M_3_ receptor; Con, control.
